# Stage-specific protein-domain mutational profile of invasive ductal breast cancer

**DOI:** 10.1186/s12920-020-00777-y

**Published:** 2020-10-22

**Authors:** Ting Yu, Kwok Pui Choi, Ee Sin Chen, Louxin Zhang

**Affiliations:** 1 Department of Mathematics, National University of Singapore, 10 Lower Kent Ridge Road, Singapore, 119076 Singapore; 2 Department of Statistics and Applied Probability, National University of Singapore, 6 Science Drive 2, Singapore, 117546 Singapore; 3 Department of Biochemistry, National University of Singapore, 8 Medical Drive, Singapore, 117596 Singapore; 4 Computational Biology Programme, National University of Singapore, 8 Medical Drive, Singapore, 117596 Singapore

**Keywords:** Cancer genes, Integrative analysis, Invasive ductal breast cancer, Stage specific genetic alteration

## Abstract

**Background:**

Understanding the mechanisms underlying the malignant progression of cancer cells is crucial for early diagnosis and therapeutic treatment for cancer. Mutational heterogeneity of breast cancer suggests that about a dozen of cancer genes consistently mutate, together with many other genes mutating occasionally, in patients.

**Methods:**

Using the whole-exome sequences and clinical information of 468 patients in the TCGA project data portal, we analyzed mutated protein domains and signaling pathway alterations in order to understand how infrequent mutations contribute aggregately to tumor progression in different stages.

**Results:**

Our findings suggest that while the spectrum of mutated domains was diverse, mutations were aggregated in Pkinase, Pkinase Tyr, Y-Phosphatase and Src-homology 2 domains, highlighting the genetic heterogeneity in activating the protein tyrosine kinase signaling pathways in invasive ductal breast cancer.

**Conclusions:**

The study provides new clues to the functional role of infrequent mutations in protein domain regions in different stages for invasive ductal breast cancer, yielding biological insights into metastasis for invasive ductal breast cancer.

## Background

Breast cancer is one of over 200 forms of cancer catalogued so far. Only lung cancer accounts for more deaths than breast cancer in women [1]. The majority of *in situ* breast cancers begin in ducts that connect the lobules to the nipple, twenty to fifty percent of which progress into invasive cancer if they are left untreated in 2016 [2]. Molecular characterizations of the malignant progression of ductal carcinoma *in situ* have been under investigation in the past two decades [3–8]. However, the mechanisms that drive progression are still unclear for ductal carcinoma *in situ* [4, 9].

Treatment options for breast cancer and prognosis depend mainly on tumor subtype and staging. The most widely used staging system classifies breast cancer into five (0 to IV) stages in terms of tumor size, whether or not the tumor has spread to the lymph nodes and the extent of metastasis [10]. Normal breast cells contain receptors that bind to the reproductive hormones estrogen and progesterone, whereas breast tumor cells may contain both, one, or neither of these receptors. Considering the presence or absence of receptors results in classification of breast cancers into four therapeutic groups in clinical settings [11]: estrogen receptor-positive (ER+), progesterone receptor-positive (PR+), HER2 amplified (HER2+) and triple negative. Perou et al. further refined this classification scheme by grouping breast cancers into four PAM50 subtypes based on the microarray expression profile of their DNA [12]: Luminal A, Luminal B, HER2-enriched and basal-like.

Individual breast cancer tumors most often carry consistent alterations of several classic oncogenes (e.g. AKT1, GATA3, PIK3CA) and tumor-suppressive genes (e.g. MAP3K1, PTEN, TP53), along with many other infrequent changes about which little is known [13–15]. Therefore, discerning which and how infrequent mutations contribute through collaboration to tumor progression is crucial [16, 17]. To meet this challenge, researchers have developed a plethora of methods for detecting cancer-associated infrequent mutations and their effects on cell signaling [18–20] (see also [21] for a review). A study along this line is expected to facilitate identification of biomarkers for early diagnosis of invasive cancers and development of combination therapies targeting specific biological pathways.

Despite the potential clinical significance, stage-specific molecular features of invasive ductal breast cancer (IDBC) remain elusive [9, 22]. Leveraging the large Phase II dataset for IDBC in The Cancer Genome Atlas (TCGA), we investigated the stage specific molecular features of this cancer. By analyzing the multi-dimensional genomic profiles, along with clinical information, from a cohort of 468 patients, we identified: (1) six novel candidate genes for IDBC (MAP2K4, ZNF384, CFBF, NOCA3, MAP3K4 and RB1), for which evidence has been absent or equivocal; (2) Src-homology 2 (SH2) domain and 21 other protein domains which carry putative driver mutations of low frequency. This study yields biological insights into malignant progression for IDBC and may assist future precision medicine efforts.

## Results

We investigated the whole-exome sequences of 468 patients with IDBC (Stage I: 87, Stage II: 297 and Stage III-IV: 92) and 112 patients with invasive lobular breast cancer (ILBC). In total, 31,242 mutations of different types were detected in 12,358 human genes (Table S1). The inter-individual mutational heterogeneity of IDBC is well indicated by the distribution of the number of genes that mutated in exactly *k* patients, which has a long low-frequency tail (Fig. S1a). More precisely, only 10 genes (TP53, PIK3CA, GATA3, MAP3K1, MUC16, KMT2C, MUC12, SPEN, MUC4) were each found to be mutated in more than 30 patients and another 20 genes in 20 to 30 patients, whereas over 10,000 genes carried mutations (including missense/nonsense base substitution, frame shift indels and four other types of mutation) in three patients or fewer.

This inter-individual mutational heterogeneity clearly suggests that there are driver mutations of low mutational frequency and these infrequent driver mutations may likely contribute through collaboration to tumor growth and progression. As such, we investigated not only novel cancer genes of low mutation frequency (Table 1), but also protein domains that were significantly altered by aggregation of infrequent mutations.

**Table 1:** Functions and mutational specificities of novel cancer-associated genes in IDBC

**Genes**	**Canonical functions**	**Cancer relevance**	**Specificity**	
			**Stage**	*In situ*
CFBF	The encoded transcription factor	Function in	Stage I	No
	regulates RUNX and other genes	aberrant estrogen		
	specific to hematopoiesis	receptor signaling		
MAP2K4,	The encoded kinases belong to a	Evading apoptosis,	No	Duct
MAP3K4	protein kinase signal transduction	Dysfunction of DNA	Stage	Duct
	cascade, which can activate the	repair mechanism		
	stress-induced P38 and JNK			
	MAPK pathways			
NCOA3	The encoded protein (AIB1)	Biomarker for	No	No
	enhances estrogen-dependent	evaluating		
	transcription	tamoxifen [24]		
RB1	RB1 prevents excessive cell growth	A tumor suppressor	No	Duct
	by inhibiting cell cycle progression	that controls cell		
	until a cell is ready to divide	growth [25]		
ZNF384	The encoded transcription factor	Involved in cell	Stage I	Duct
	involved in extracellular matrix	proliferation in		
	remodeling	other cancers		

### Cancer genes carrying stage-specific mutations in IDBC

MutSigCV2 reported 12 out of 12,358 mutated genes at a false discovery rate (FDR) with *q*-value ≤0.1 (Table S2), which examines the gene-specific abundance of mutations relative to the background mutation rate, mutation conservation and clustering [18]. Among these were six functionally established cancer genes AKT1, GATA3, MAP3K1, PIK3CA, PTEN and TP53 (for each *q*-value ≤10^−13^). The other six reported genes that have less clear functions in breast cancer are CBFB, MAP2K4, MAP3K4, NCOA3, RB1 and ZNF384, whose biological functions and cancer relevance are summarized in Table 1. CBFB and ZNF384 are cancer genes for leukemia; RB1 is a gene for retinoblastoma, sarcoma and small-cell lung cancers and MAP3K4 is a gene for pancreatic, breast and colorectal cancers, as listed on the Cancer Gene Census [23]. Mutations that occurred in these genes are illustrated in Fig. S2 and Fig. S3.

All except for NCOA3 (4.9% versus 4.9%) and CBFB (2.56% versus 2.68%) mutated significantly more frequently in IDBCs than in 112 patients with ILBC: MAP2K4 (4.7% versus 1.6%, *P*-value <10^−5^), RB1 (4.1% versus 0.8%, *P*-value =1.5×10^−8^), ZNF384 (4.3% versus 1.6%, *P*-value = 9.2×10^−5^) and MAP3K4 (2.3% versus 1.2%, *P*-value =0.028), neither of which were reported by MutSigCV2 when only 112 ILBC genomes were used.

**Fig. 1:**
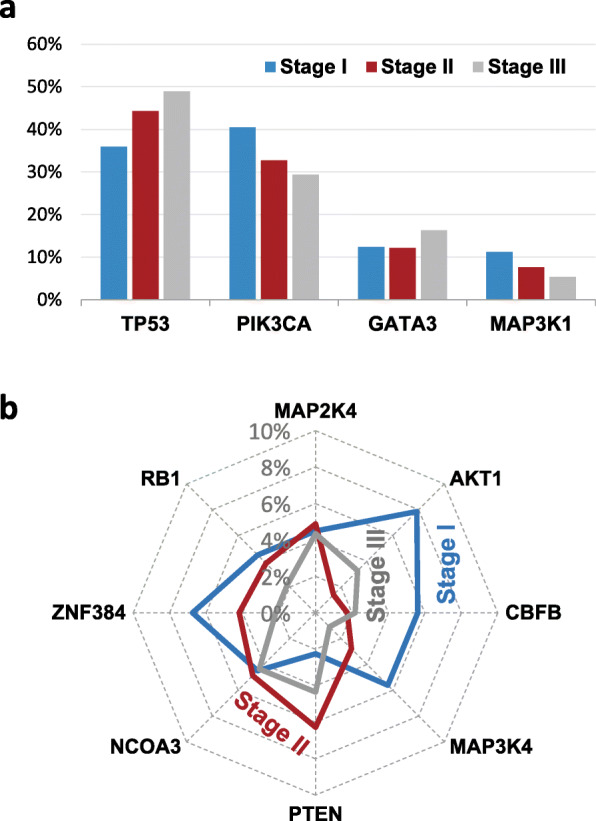
The mutational profiles of significantly mutated genes in IDBC. (**a**) The mutation coverages (MCs) of the four significantly mutated genes with a mutation frequency > 9% in different stages. (**b**) The MCs of the eight significantly mutated genes with a mutation frequency < 9% in different subtypes

The mutation coverage (MC) of a gene is the percentage of patients carrying mutations in the gene, also called mutation prevalence [26]. The MCs did not vary much across the stages for TP53, PIK3CA, GATA3, and MAP3K1 (Fig. 1a). However, the MCs of AKT1, CBFB, MAP3K4 and ZNF384 in Stage I were at least 1.5 times as high as in other stages, having *P*-values of 5.27×10^−3^, 2.7×10^−2^, 5.09×10^−2^, 4.02×10^−2^ for enrichment in Stage I (Fig. 1b).

### protein domains were recurrently hit by infrequent mutations

Protein domains are evolutionarily and structurally conserved units with specific functions in protein sequences. Tallying mutations in the domain-coding regions in all genes containing a specific protein domain allow us to identify mutations that are rare at the gene level, but occur recurrently within the protein domain [27, 28]. Therefore, a domain-based approach enables the discovery of new alterations that confer functional advantage to cancer cell development but are not detected through gene-by-gene analysis.

**Fig. 2:**
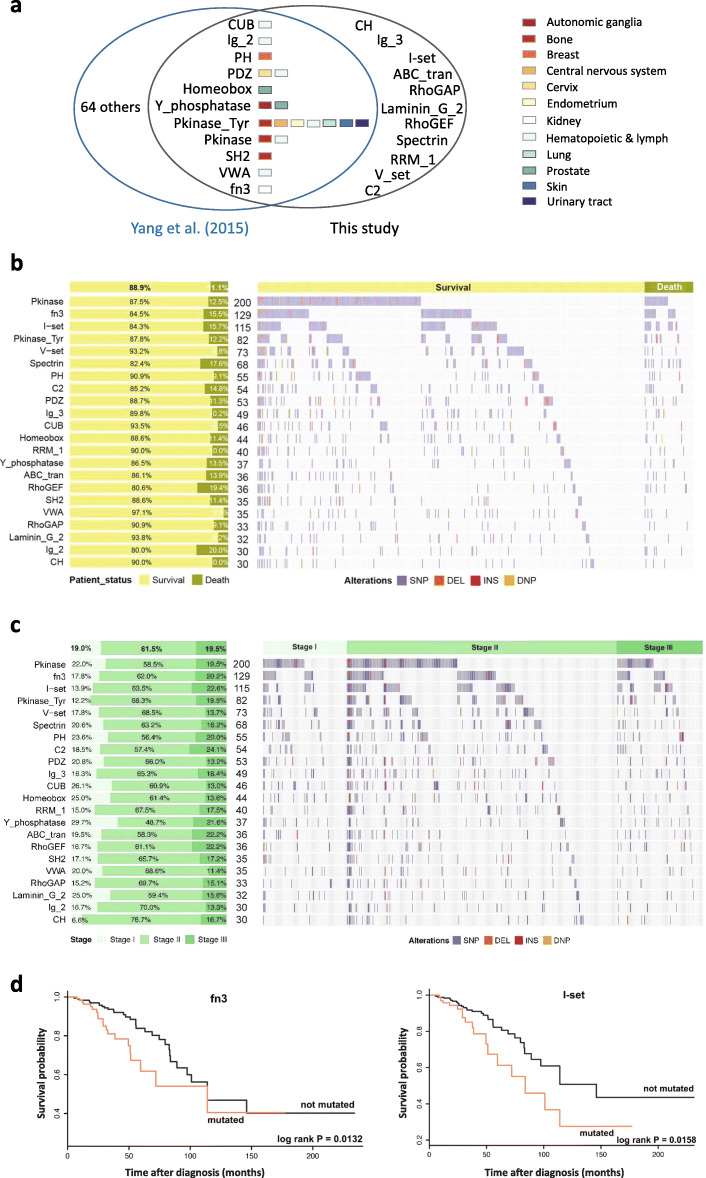
22 significantly mutated protein domains and their mutational spectra. (**a**) 11 reported protein domains were also identified by Yang et al. [28] for other cancers. Here, “others” represent those protein domains that were only reported in [28]. (**b**) and (**c**) The distributions of mutations in the 22 protein domains in terms of survival status and staging. *Left panel*: The percentage of surviving (and deceased) patients (resp. patients in a stage) in which each protein domain is mutated. *Right panel*: The distribution of the four types of mutations in the groups of patients for the 22 protein domains. *Middle column*: The number of patients in which a protein domain is mutated. Of note, none of these protein domains was mutated in the patients listed at the end of each group. SNP, single nucleotide polymorphism; DEL, deletion; INS, insertions; DNP, double nucleotide polymorphism. (**d**) The Kaplan-Meier survival analysis for protein domains fn3 and I-set

In the 468 patients with IDBC, 4,793 mutations occurred in genomic sequences that encompass the encoding regions of 1,217 (PfamA) protein domains in 3,625 genes (Table S3). 22 protein domains were found to have mutations being distributed in at least 30 genomes (6.4%), resulting in a *P*-value of less than 0.05 and a normalized Shannon entropy of at least 0.71 (Fig. S4). 18 of these protein domains are found in cancer genes (based on the Cancer Gene Census [23]), whereas supporting evidences for involvement in breast cancer have been reported for the other four in the literature [29, 30]. In addition, 11 of these protein domains were also reported by Yang et al. in their integrative analysis of pan-cancer genomes [28] (Fig. 2a). (These 22 protein domains are functionally annotated in Table S4.)

The mutational spectra of the 22 protein domains were examined in terms of survival status (Fig. 2b) and stages (Fig. 2c). Mutations in both the I-set and fn3 domains are enriched in the group of patients who died with *P*-values of 0.056 and 0.047, respectively. Additionally, they were depleted in Stage II (*P*-values <2.2×10^−16^). The Kaplan–Meier survival analysis further showed that mutation in these two protein domains agree with the poor survival outcomes (cutoff *P*-value = 0.05, log rank test, Fig. 2d), as well as Spectrin, Ig_2, and RhoGEF domains (Fig. S5). The hazard ratio (HR) analysis also suggests this fact. HR of I-set and fn3 are 2.0473 and 2.0711, respectively, corresponding to *P*-values 0.0144 and 0.0112.

### Critical mutations in SH2 domains

Of the 22 protein domains, Pkinase, Pkinase_Tyr, Y-Phosphatase and Src-homology 2 (SH2) play important roles in protein tyrosine kinase (PTK) signaling pathways. The Pkinase domains were found to be mutated 374 times in 186 kinase genes in 200 patients. The Pkinase_Tyr domain mutated 115 times in 67 PTK genes, including 10 cancer genes [23]. Mutations within the Pkinase_Tyr domain were also found recurrently in breast and seven other cancers. Together with tyrosine kinases, 107 protein tyrosine phosphatases (also called Y-phosphatases) regulate a plethora of cellular processes, including cell growth and oncogenic transformation. Therefore, it is not surprising that the Y-phosphatase domain were also found to highly mutate in the 468 patients.

**Fig. 3:**
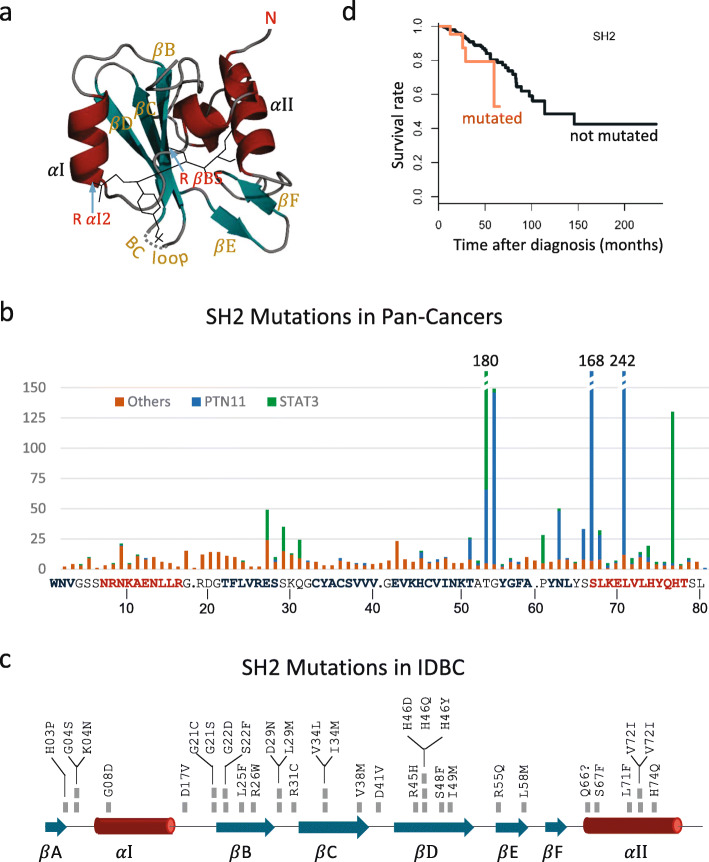
SH2 mutations in IDBC and pan-cancers. (**a**) The folded SH2 comprises a central anti-parallel sheet, consisting of the three *β*-strands (*β*B, *β*C, and *β*D, cyan), sandwiched between two *α*-helices (*α*I and *α*II, dark red). The bound phosphopeptide (thin dark lines) straddles the sheet in such a manner that the phosphotyrosine (pY) binding pocket lies on one side, whereas the binding pocket for the residues in the positions C-terminal to pY lies on the other side. The two R residues in *α*I (R *α*I2) and in *β*B (R *β*B5) make key contact with pY. This ribbon representation is redrawn based on the folded structure of the C-terminal SH2 domain in P85 *α* (PDB accession number: 1QAD). N, N-terminal end. (**b**) The positional distribution of SH2 mutations in pan-cancers. Analysis was made based on cancer genome data downloaded from the COSMIC database ([33], accessed 20 August 2017) and sequence alignment of the 23 SH2 domains having mutations in the 468 patients (Supplementary Document, page 15). The horizontal axis represents the peptide sequence containing the first 80 amino acids of the C-terminal instance of the SH2 domain in P85 *α*. Structural elements are colored in the same way as in Panel a. (**c**) The SH2 mutations occurring in the 468 patients are from 23 proteins (Table S5). (**d**) The Kaplan–Meier survival analysis for the SH2 domain. The log rank *p*-value for 0.173

SH2 domains are critical mediators found in 96 functionally different human proteins [31]. SH2 mutations could lead to constitutive dysfunction of kinase/SH2 domains or the upstream and downstream rewiring of the PTK signaling pathways, as outlined by Creixell et al. recently [16]. The folded structure of SH2 consists of a central anti-parallel three-stranded *β*-sheet flanked by two *α*-helices (Fig. 3a); each instance of SH2 binds phosphotyrosine-containing sequences with high affinity and specificity with two binding pockets [32]. Two R residues, R *α*I2 and R *β*B5, play particularly crucial roles in phosphotyrosine binding with further interactions with three residues at Positions *β*D2 to *β*D4 (Fig. 3b). Here, we examined SH2 mutations in pan cancers available in the COSMIC database [33]. Our pan-cancer analysis showed that mutation rarely occurred at *α*I2 and *β*B5 but recurrently occurred at one position C-terminal to them, as well as at the first position in the BC loop. For IDBC, a PTPN11 mutation occurred at *α*I2; two mutations in STAT4 and SRMS occurred at *β*B4 and *β*B5, respectively; and three mutations in ABL2, P85 *α* and TEC occurred at *β*D4.

The binding pocket for residues that lie three to five amino acids C-terminal to the pY involves residues on the opposite face of the central sheet, the EF turn and *α*II (Fig. 3a). The PTPN11 and STAT3 mutations recurrently occur near this pocket. For IDBC, five mutations in ABL2, P85 *β*, PLC *γ*2, SHD and SRMS were found in *α*II (Fig. 3c). Since SH2 must obtain sufficient binding energy from the recognition of nearby residues to achieve a high degree of binding specificity, these identified mutations are likely to affect the binding affinity of SH2 and hence to have critical roles in IDBC, as exemplified by BTK [34] and PTPN11 [35].

### Alteration of ERBB2 signaling pathway is beyond HER2

SH2 mutations could lead to constitutive dysfunction of kinase/SH2 domains or the upstream and downstream rewiring of the PTK signaling pathways. To validate whether PTK signaling pathways were likely interrupted or not, we conducted a network analysis of the mutational and amplification profiles of IDBC using the HotNet2 program [19]. Our network analysis identified 11 protein complexes and signaling pathways that were significantly mutated (Table S6), including the ERBB2 signaling pathway, the roles of which in breast cancer have been studied extensively. The ERBB2 signaling pathway were altered by infrequent mutations aggregately (Fig. S6).

## Discussion

Cancer is intimately associated with mutations in cancer genes. Because of the vast heterogeneity of breast tumors [13], characterizing genetic alterations associated with the malignant progression of cancer cells remains a great challenge. We set out to resolve this issue for IDBC by investigating stage-specific genetic alterations caused by infrequent mutations using 468 whole-exome sequences and related clinical information. Our study of genetic alterations complements well recent studies of the gene expression signatures of IDBC [3–5, 22] and the molecular portraits of invasive lobular breast cancer [36]. It yields biological insights into the malignant progression of IDBC and reveals potential new avenues for seeking targeted agents in breast cancer.

We found six novel candidate genes of low mutation rate for IDBC: CBFB, MAP2K4, MAP3K4, NCOA3, RB1 and ZNF384, for which evidence has been absent or equivocal. Infrequent mutations in CBFB, MAP3K4 and ZNF384 are enriched in Stage I, together with classic breast cancer gene AKT1 (Fig. 1b). This suggests the hypothesis that these four genes of low mutation rate play Stage I-specific roles, whereas classic cancer genes MAP3K1, PTEN, TP53, GATA3 and PIK3CA are important in the entire malignant progression of IDBC.

We further identified 22 recurrently mutated protein domains by tallying infrequent mutations across the patients, most of which are contained in the genes in the Cancer Gene Census. These protein domains include the Pkinase, Pkinase_Tyr, Y-Phosphatase and SH2 domains that play important roles in the PTK signaling pathways. In particular, SH2 mutations could lead to constitutive dysfunction of kinase/SH2 domains or the upstream and downstream rewiring of the PTK signaling pathways, as outlined by Creixell et al. recently [16]. Therefore, our investigation of SH2 mutations facilitates identification of new biomarkers and new drug targets among 96 SH2-containing proteins.

## Conclusions

Our integrative analyses provide biological insights into the progression from *in situ* ductal carcinoma to metastatic cancer. It may assist determining biomarkers for early detection of IDBC, which can be a deciding factor between successful treatment and death. It can also be valuable in designing combinatorial therapeutic treatments defined at the network level for IDBC [37].

## Methods

### Data collection

The datasets for 468 patients with IDBC and 112 patients with ILBC were used in this study, which were deposited in the Phsae II of the TCGA project (http://tcga-data.nci.nih.gov/tcga/dataAccessMatrix.htm, download 1 March 2016). These datasets contain information on mutation (single nucleotide variants and copy number variants) and survival status of tumors (Table S1). The 468 patients with IDBC were originally staged using different versions of the American Joint Committee on Cancer TNM system in the past decade [10]. To keep consistency in stage information, we re-staged the cases using the seventh edition of the manual if they were staged using the sixth or earlier edition of it. Additionally, there were only 10 patients in Stage IV and thus we merged Stage IV into Stage III, obtaining 89, 287, and 92 cases in Stages I, II and III, respectively (Table S1).

### Survival analysis

The SRUVIVAL R-program was used for the Kaplan–Meier and COX–PH analyses. The two survival analyses are fully documented in the Supplementary Document. Of note, *P*-value was computed by using the log-rank test in the Kaplan–Meier analysis and the Wald test in the Cox-PH analysis.

### Prioritization of mutated genes

MutSigCV2 (Lawrence et al., 2014) was used to identify a list of 12 recurrently mutated genes discussed in the “Results” section. MutSigCV2 uses genomic covariates to account for the specific background mutation rate for each gene and uses both *P*-values and *q*-values for measuring the mutation significance. Like any bioinformatic tool for mutational analysis in cancer biology, the output from a MutSigCV2 analysis is sensitive to the input dataset [38]. We conducted a kind of saturated analysis [18], like bootstrap analysis, for further reducing false positive predictions. We created six extra datasets by randomly selecting 410, 420, 430, 440, 450 and 460 patients from the total of 468 patients. We then ran the program on the original dataset as well as the six derived ones, resulting in the final list of 12 significantly mutated genes via consensus (Table S2; Section 3.1 of the Supplementary Document).

### Mutational analyses of mutated protein domains

Protein domain analysis was conducted using a method similar to the one in [27]. Mutations in the protein domain-coding regions in all genes containing a given protein domain are tallied to identify mutations that are rare at the gene level, but occur recurrently within the protein domain. Missense mutations were mapped onto the Pfam human protein domains (Pfam-A database, version 29; https://doi.org/ftp://ftp.ebi.ac.uk/, accessed 1 March 2017) (Fig. S2). The Human Pfam-A database contained 49,636 protein domains, 33,044 (sequence) families, 739 motifs, 9,238 repeats, 240 coiled-coils and 192 disorders. Only protein domains with E-values <10 e-5 were used in our analysis. In total, 1,217 Pfam-A protein domains had mutations across 468 patients (Table S3).

We made two assumptions: (i) a mutation is uniformly distributed in each protein gene (i.e., a mutation hits the encoding region of a protein domain with a probability equal to the ratio of the domain length to the protein length and (ii) a mutation occurs independently in each position of a gene sequence. Under the two assumptions, the number *N* of mutations occurring in the domain-encoding regions of the respective gene members has a Poisson-binomial distribution, for which the exact *P*-value of the event that *N*>*#*(*observed* *mutations*) can be computed with a dynamic programming algorithm. Our computer program developed from this method is available upon request. It gave *P*-values that were more accurate than the permutation test [27], particularly when its true value was less than 10^−7^.

### Analysis of network and pathway alterations

We used the HotNet2 program [19] to identify significant alterations in signaling pathways and protein complexes. HotNet2 was designed using an insulated heat diffusion process, which is actually a random walk with a restart, on a protein network to capture the mutation alterations within a local subnetwork around a protein.

## Supplementary information

**Additional file 1** Supplementary document for data analyses.

**Additional file 2** Fig. S1-S6.

**Additional file 3** Table S1: (Related to Fig. 1) The mutational and clinical information for 468 patients.

**Additional file 4** Table S2: (Related to Table 1) The gene lists output from MutSigCV2.

**Additional file 5** Table S3: (Related to Fig. 2) The 1,217 domains that mutated in IDBC.

**Additional file 6** Table S4: (Related to Fig. 2) Functional annotation of the identified 22 protein domains.

**Additional file 7** Table S5: (Related to Fig. 2) The identified 22 protein domains.

**Additional file 8** Table S6: 11 protein complexes and signaling pathways identified by network enrichment analysis.

## Data Availability

Data sharing is not applicable to this article as no datasets were generated or analysed during the current study.

## Abbreviations

COSMIC: ■■■
ER+: estrogen receptor-positive
FDR: false discovery rate
HR: hazard ratio
HER2+: HER2 amplified
IDBC: invasive ductal breast cancer
ILBC: invasive lobular breast cancer
MC: mutation coverage
PR+: progesterone receptor-positive
PTK: protein tyrosine kinase
SH2: Src-homology 2
TCGA: The Cancer Genome Atlas
