# Supplementary material for: Stage-specific protein-domain mutational profile of invasive ductal breast cancer
Source: BMC Med Genomics. 2020 Oct 22;13(Suppl 10):150. doi: 10.1186/s12920-020-00777-y (PMC7580001; doi:10.1186/s12920-020-00777-y)
Supplement: Supplementary file 1 — Additional file 1 Supplementary document for data analyses. [file 12920_2020_777_MOESM1_ESM.pdf]

Supplementary Document

on

Integrative Analysis Identifies Subtype/Stage-Specific Alterations of Pathways and Protein Complexes for Invasive Ductal Breast Cancer

By Yu et al.

1. Summary of the study · page 2
2. Data collection · page 3
3. Methods · page 4
   3.1. Identification of cancer-associated genes page 4
   3.2. Recurrently mutated protein domains page 6
   3.3. The Kaplan–Meier survival and hazard ratio analysis page 8
4. Analyses of significantly altered protein domains · · · · · · · · · · · · · · · · page 9
   4.1. Twenty-two altered protein domains page 9
   4.2. Pan-cancer analysis of SH2 mutations page 10
5. References · page 13

1. Summary of the study

In this study, we attempt to identify stage/subtype-specific cancer-associated alterations in genes, protein domains, signaling pathways and protein complexes in invasive ductal breast cancer (IDBC). We analyzed the exome sequences of 468 patients with IDBC using a framework made form MutSigCV2 (Lawrence et al., 2013), mutational entropy (Miller et al., 2015). The framework also contains novel statistic methods for evaluating the significance of gene-associated genes and domains of low mutation frequency.

We identified 12 cancer gene candidates, 22 cancer-associated protein domains and 11 alterations of signaling pathways and protein complexes that have potential for developing combinatorial drug targets and treatments. Six of the identified genes are classic oncogenes or tumor suppressors, including TP53, PI3KCA and PTEN, and the remaining six are novel cancer-associated genes for which evidence was previously absent or equivocal. The cancer-related functions of these alterations were investigated through database mining and literature study. Their clinical relevance is examined via a Kaplan–Meier survival analysis.

2. Data collection

We analyzed the mutational profiles of 468 in situ ductal carcinomas published by Ciriello et al. (2015) and available on the TCGA data portal. The tumor samples were taken from 468 patients with invasive breast adenocarcinomas who underwent surgical resection without any prior treatment (e.g., chemotherapy or radiotherapy) as described in The Cancer Genome Atlas (2012). The 468 tumors were typed as: basal-like (109), Luminal B (119), Luminal A (193) and HER2 (47) (Supplemental Table S1). The stage and survival information were downloaded from the TCGA data portal (https://gdc-portal.nci.nih.gov/, accessed 5 June 2015).

Our pan-cancer analyses of somatic mutations in the SH2 domain and blood factor Protein S were conducted using the mutation data downloaded from the COSMIC database of mutations in cancer (http://cancer.sanger.ac.uk/cosmic, accessed 10 August 2017).

Breast cancer is staged using the American Joint Committee on Cancer (AJCC) stage system (American Joint Committee on Cancer, 2010), which is based on: (a) the size of the breast tumor (T), (b) whether the cancer has spread to nearby lymph nodes (N) and (c) whether the cancer has metastasized (M). This so-called TNM-based stage system has three recent versions: the fifth, sixth and seventh editions. To maintain consistent stage information, we restaged the cases with the seventh edition if they were staged with an earlier version

The AJCC staging system classifies breast cancer into four stages and further divides a stage into sub-stages. Like most genetic studies, we only considered four main stages. We further merged Stage IV into Stage III, as there were 10 cases in Stage IV, which was too small to be statistically analyzed. Therefore, The analyzed data comprises of 89, 287 and 92 cases in Stages I, II and III, respectively (Table S1).

3. Methods

We conducted an integrative analysis using a framework consisting of three published programs: MutSigCV2 (Lawrence et al., 2013), mutational entropy (Miller et al., 2015) and several novel statistical methods for measuring the significance of cancer-associated genes and protein domains of low mutation frequency.

3.1 Identification of cancer-associated genes

MutSigCV2 considers different genomic covariates when calculating the gene-specific background mutation rate. It measures mutational significance using both the *P*-value and the *q*-value. The *P*-value represents the possibility that the observed mutation count for a specific gene exceeds the count expected by chance, whereas the *q*-value is obtained from the *P*-value by considering the false discovery rate. The files (e.g., exome_full192.coverage.txt, gene.covariates.txt, mutation_type_dictionary_file.txt and chr_files_hg19) that were used to run MutSigCV2 were downloaded from the Broad Institute (http://archive.broadinstitute.org/cancer/cga/mutsig_run, accessed January 2016).

Using the MutSigCV2, we identified 24 genes using the *q*-value < 0.1 (Table S2). Among these 24 genes, some have small mutation numbers. For example, three mutations occurred in *ACOT4*, two in *KCNE4*, five in *MAGEA8*, nine in *MEF2A* and six in *SARM1*. This fact indicates the possibility of false positive predictions. To make a robust analysis, we randomly chose 410, 420, 430, 440, 450 and 460 patients from the entire group of patients and then re-ran the MutSigCV2 on each of these random datasets. Finally, we obtained seven lists of significant mutated genes (Table S2). We further selected 12 significantly mutated genes each with *q*-values of < 0.1 and > 10 mutations by weighing genes in the seven lists. These are *AKT1*, *GATA3*, *MAP3K1*, *PIK3CA*, *PTEN*, *TP53*, *CBFB*, *MAP2K4*, *RB1*, *NCOA3*, *MAP3K4* and *ZNF384*.

Detection and visualization of mutation-depleted and -rich regions in proteins. Vitamin K-dependent Protein S is a cofactor of the anticoagulant enzyme-activated protein C in a coagulation cascade. It was identified as a key component of a signaling pathway that was altered by rare mutations in our network and pathway analysis. The mutation data from the COSMIC database (accessed 20 August 2017) indicates that 264 somatic mutations have been detected in different cancers. We apply the run statistics to detect and visualize mutation rich

regions under the assumption that these mutations occur independently in 676 amino acids of Protein S each with probability (i.e., 264/676).

More specifically, we drew a sliding window plot (Figure 4C), in which peaks indicate mutation clusters, whereas deep valleys indicates mutation sparse regions. The statistical significance of the peaks observed in the sliding window plot were measured according to the distribution of the maximum number of mutations, W^*, over all windows. The upper tail probability, $P(W^* \geqslant k)$, was used to justify the significance of a mutation cluster, which was estimated by a simulation with 10 million repetitions.

Radar chart. We used radar charts to represent the mutational features of the subtypes and stages on the selected genes with low mutation frequency rates. We counted the mutation numbers of the genes for each subtype/stage. We then normalized the mutation numbers by dividing by the total number of patients for each subtype/stage. For example, the TP53 gene mutated in 26 tumors in the Luminal A subtype and we thus obtained a coverage rate of 26/193 (13.5%), where 193 is the total number of the patients with the Luminal A subtype in the dataset. These subtype/stage-specific percentages were used to draw the two radar charts in Figure 1.

3.2 Recurrently mutated protein domains

Mapping mutations to protein domain regions. The Human Pfam-A database (version 29, 9606.tsv.gz, ftp://ftp.ebi.ac.uk/pub/databases/Pfam/releases/Pfam29.0/proteomes/, accessed 17 August 2017) was downloaded in May 2016. It contains 49,636 protein domains, 33,044 (sequence) families, 739 motifs, 9,238 repeats, 240 coiled-coils and 192 disorders. Only protein domains with E-values < 10 e–5 were used in our analysis.

After the Uniprot accession number was converted to HGNC gene symbol using the human gene file GRCh38.p7 (http://asia.ensembl.org/biomart/martview/6f709a94f4b24689bd2135ea7d59b544, accessed 17 August 2017), missense mutations were mapped onto Pfam domain sequences using the gene symbols, the positional information on mutations and the domains as shown in Figure S7. In total, 6793 mutations occurred in the sequences of 1217 human Pfam-A protein domains (Table S3).

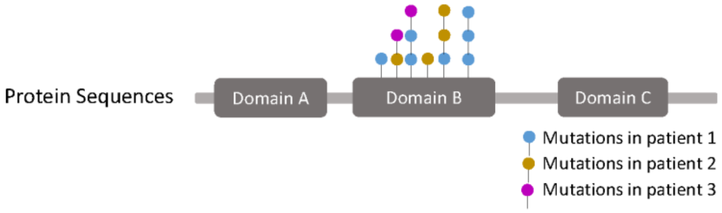

Figure S7 | Mapping mutations occurring in patients to the domain-encoding regions.

Significance assessment for protein domain mutation. To assess whether a mutation in a protein domain occurred statistically more often than what would be expected by chance, we made the following assumption:

> *The number of mutations occurring in an instance D_g of a protein domain D follows a binomial distribution with the parameters n_g and $p(n_g, D_g)$. Here, n_g is the total number of mutations occurring in g and $p(n_g, D_g)$ is the probability that a mutation hits the instance of D in the gene, which is equal to the ratio of the length of D_g to the length of the entire gene g.*

We also assume that mutations occur independently in different genes in the domain-containing gene family and that the total number N of mutations occurring in the domain follows a Poisson binomial distribution. The P-value of the event that k or more mutations were observed in the domain was computed with a dynamic programming algorithm (Chen and Liu, 1997). Our

computer program is available upon request. It gave more accurate *P*-value than the permutation test (Miller et al., 2015), particularly when its true value was less than 1 e–2.

Computation of the mutation entropy for protein domains. Shannon information entropy was used to measure the extent to which the mutations that occurred in a specific domain were spread across a large set of domain instances in genes. Let F be the set of all the genes containing D for a protein domain D and let m_g mutations occur in the instance of D in gene g. The entropy of domain mutation for D (Miller et al., 2015) is then defined as:

$$\bar{S}_D = -\sum_{g\in F} \frac{m_g}{\sum_{t\in F} m_t} \log \frac{m_g}{\sum_{t\in F} m_t}.$$

The entropy score is between 0 and log (|F|). In particular, if only one gene has mutations in D, then $\bar{S}_D = 0$; if all the genes in F mutate the same number of times, then $\bar{S}_D = \log\ (|F|)$. To facilitate the comparison of entropy scores for domains with different sized protein domain families, we normalized this entropy score by dividing it by the maximum mutational entropy value, log (|F|).

3.3 The Kaplan–Meier survival analysis and the hazard ratio analysis

Survival analysis is often complicated when patients drop out or die during the study period. The Kaplan–Meier survival analysis is a nonparametric method widely used to estimate the survival probability as a function of time after treatment in clinical cancer studies. It provides a graphical estimate of the survival function.

The Kaplan–Meier survival curve is defined as the probability of surviving in a given period of time, considering time in many small intervals (Kleinbaum and Klein, 2012). The survival probability p_t at a time point t is approximated as:

$$p_t = 1 - \#(\text{patients died before the time point } t)/\#(\text{patients living at the start}).$$

The Cox Proportional Hazards (PH) model is the most commonly used regression model relating the survival time of an individual with his or her covariates. In this paper, we ran the R package SURVIVAL for Kaplan–Meier analysis and BRESLOW for the Cox PH model-based analysis (Therneau, 2011). The P-value was computed from the log-rank and the Wald tests for the Kaplan–Meier and the Cox PH model analyses, respectively.

4. Analyses of significantly altered protein domains

4.1 Twenty-two altered protein domains

Our study identified 22 protein domains that not only significantly mutated but also had high mutational entropy in IDBC. Half of them have been reported in a similar study by Yang et al. (2015) (Figure S8).

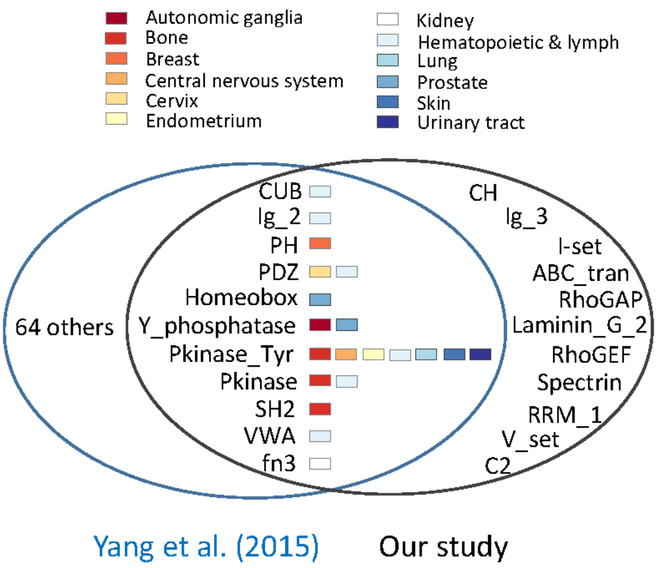

Figure S8 | Eleven protein domains that were also identified for other cancers in a pan-cancer analysis (Yang et al., 2015). The protein domains only reported by Yang et al. are omitted here.

These 22 protein domains are divided into eight groups.

(1) Pkinase (PF00069), Pkinase_Tyr (PF07714), Y-Phosphatase (PF00102) and SH2 (PH00017)

These four protein domains contribute to the protein tyrosine kinase (PTK) signalling pathways. Pkinase had as many as 347 mutations in 186 kinase genes in 200 patients. Pkinase_Tyr is a kinase domain only appearing in 90 PTKs. It mutated 115 times in 67 PTKs in the patients. Together with tyrosine kinases, 107 protein tyrosine phosphatases regulate a plethora of cellular processes, including cell growth and oncogenic transformation. It is not surprising that Y-Phosphatase (PF00102) also frequently mutated. SH2 (PH00017) appears in adaptor or adaptor-interacting proteins, which facilitate signal flow in a receptor PTK signalling pathway.

(2) Spectrin (PF00435) and CH (PF00307)

The two protein domains appear in a group of giant proteins in the cytoskeletal structure.

(3) C2 (PF00168), PDZ (PF00595) and PH (PF00169)

They are involved in binding or anchoring proteins to cellular membranes to interact with other components of cancer-related signal transduction pathways. The cancer genes *PTEN* and *PI3K* have the C2 domain.

(4) VWA (PF00092), Laminin_G_2 (PF02210), fn3 (PF00041) and CUB (PF00431)

These four domains appear in extracellular proteins participating in cell adhesion, angiogenesis, migration and signaling. For example, Protein S (PS) discussed in the Results section contains a Laminin_G_2 domain.

(5) ABC_tran (PF00005)

This is an ATP-binding domain appearing, together with a transmembrane domain, in the ABC transporters. The transmembrane ABC transporters are mainly responsible for the export of extracellular toxins in humans.

(6) RhoGEF (PF00620) and RhoGAP (PF00621)

The former is a structural domain of the guanine nucleotide exchange factors that activate Rho/Rac/Cdc42-like GTPases by stimulating the release of guanosine diphosphate (GDP), whereas the latter is a structural domain of GTPases that activate the proteins bound to the activated G proteins to terminate a signaling event.

(7) Homeobox (PF00046) and RRM_1 (PF00076)

The former is a DNA-binding domain found in the homeobox proteins. The latter is a RNA recognition motif in some RNA-binding proteins.

(8) I-set (PF07679), V_set (PF07686), Ig_2 (PF13895) and Ig_3 (PF13927)

These three domains mainly appear in immunoglobulin proteins. However, they can also appear in receptor PTKs and certain proteins in the cytoskeletal structure.

4.2 Pan-cancer analysis of the SH2 mutations

Point mutations occurred in the 23 SH2 domains (Table S8) in the 468 patients with IDBC. SH2 mutations in the 23 SH2 domains were mapped onto the consensus sequence of the SH2 domain using a multiple sequence alignment made with CLUSTAL O(version 1.2.4) (Figure S9). The same mapping was also used for annotating SH2 mutations downloaded from the COSMIC databases.

Table S8. Twenty-three SH2 domains mutated in the 468 patients with IDBC

Proteins	UniProt ID	SH2 Mutations	Proteins	UniProt ID	SH2 Mutations
ABL2	P42684	H219D, I222M, L244F	SHD	Q96IW2	V313I
BCRA3	O75815	L180M	SHE	Q5VZ18	L454M
BMX	P51813	D341? (frame_shift_ind)	SOCS6	O14544	S405F, S453F
BTK	Q06187	R332H	SRMS	Q9H3Y6	R147Trp
INPPL1	O15357	Q93?	STAT1	P42224	D593V
ITK	Q08881	K242N	STAT3	P40763	V619L, S636F
PIK3R1	P27986	G644C, G644S, H668Q	STAT4	P14765	L597F
PIK3R2	O00459	H404R	STAT5A	P42229	R649Q
PLCG2	P16885	V717I	STAT6	P42226	D565N, I571M
PTPN11	Q06124	G13D	SYK	P43405	V55M
SH2B3	Q9UQQ2	G367S, R398C	TEC	P42680	G269D
SHB	Q15464	H412P			

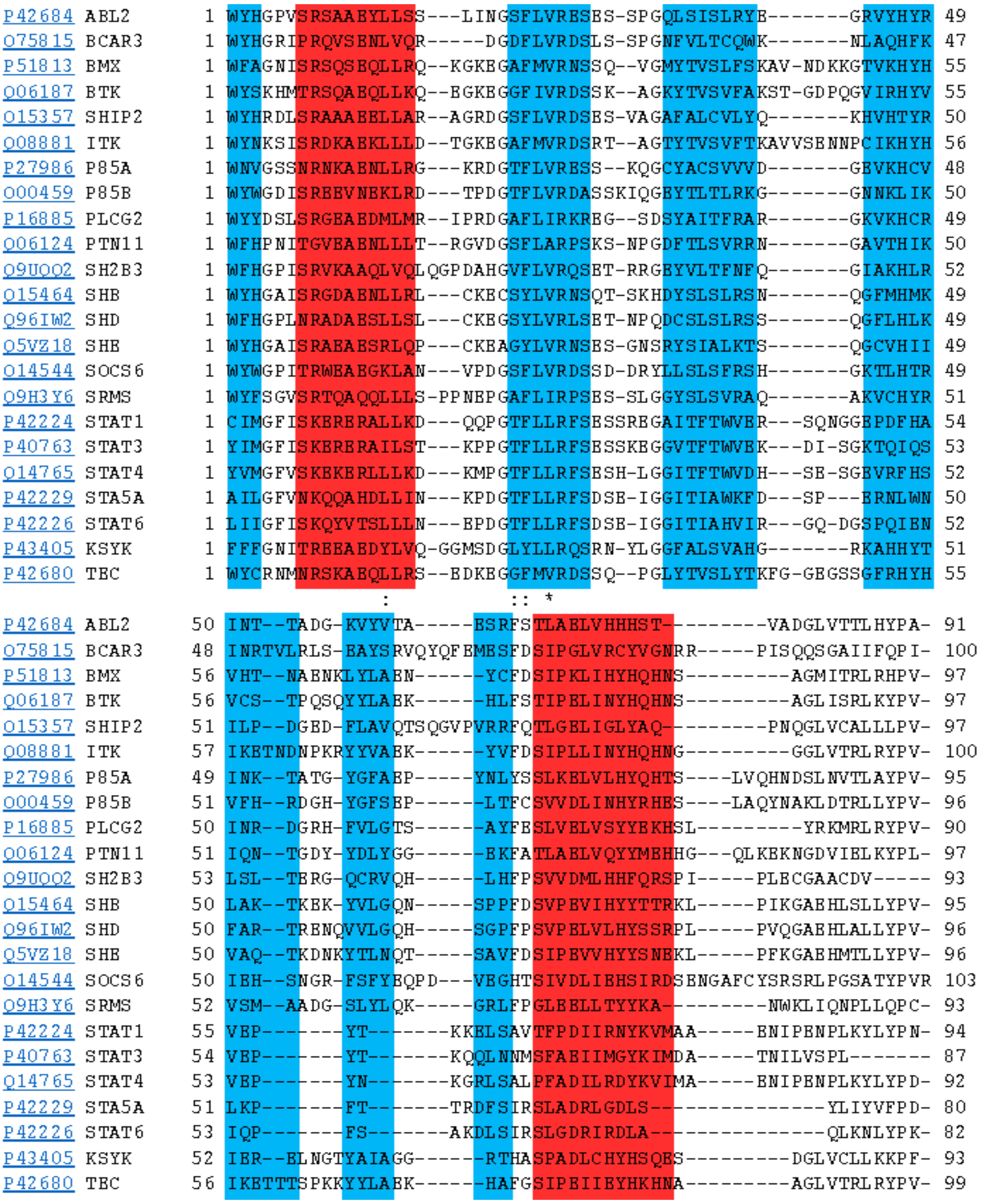

Figure S9. Structural alignment of 23 SH2 domains that mutated in the 468 patients with IDBC. It was obtained with CLUSTAL O(version 1.2.4).

References

1. American Joint Committee on Cancer, 2010. Cancer Staging Manual (edition 7). Springer-Verlag, New York, USA.
2. Chen, S.X., Liu, J.S., 1997. Statistical applications of the Poisson-Binomial and conditional distributions. Statistica Sinica, 7(4): 875-892.
3. Ciriello, G., Gatza, M.L., Beck, A.H., et al., 2015. Comprehensive Molecular Portraits of Invasive Lobular Breast Cancer. Cell, 163(2): 506-519.
4. Kleinbaum, D.G., Klein, M., 2012. Survival Analysis: A Self-Learning Text. Third. Statistics for Biology and Health. Springer, New York, USA
5. Lawrence, M.S., Stojanov, P., Polak, P., et al., 2013. Mutational heterogeneity in cancer and the search for new cancer-associated genes. Nature, 499(7457): 214-218.
6. Miller, M.L., Reznik, E., Gauthier, N.P., et al., 2015. Pan-Cancer Analysis of Mutation Hotspots in Protein Domains. Cell Syst, 1(3): 197-209.
7. The Cancer Genome Atlas, 2012. Comprehensive molecular portraits of human breast tumours. Nature, 490(7418): 61-70.
8. Yang, F., Petsalaki, E., Rolland, T., Hill, D.E., Vidal, M. and Roth, F.P. (2015). Protein domain-level landscape of cancer-type-specific somatic mutations. PLoS Comp. Biol. 11, art. e1004147.
9. Therneau, T. and original Splus−>Rport by Thomas Lumley (2011). survival: Survivalanalysis, including penalised likelihood.Rpackage version 2.36-10.http://CRAN.R-project.org/package=survival.
