# Supplementary material for: Stage-specific protein-domain mutational profile of invasive ductal breast cancer
Source: BMC Med Genomics. 2020 Oct 22;13(Suppl 10):150. doi: 10.1186/s12920-020-00777-y (PMC7580001; doi:10.1186/s12920-020-00777-y)
Supplement: Supplementary file 2 — Additional file 2 Fig. S1-S6. [file 12920_2020_777_MOESM2_ESM.pdf]

Stage-specific protein-domain mutational profile of Invasive Ductal Breast Cancer

By Yu et al.

Supplemental Data Items

Figure S1 The mutational heterogeneity of IDBC in terms of cancer subtypes
Figure S2 Mutations in AKT1, PIK3CA, CBFB and MAP2K4
Figure S3 Mutations in MAP3K4, NCOA3, RB1 and ZNF384
Figure S4 The P-value is plotted against the Shannon entropy for 1,217 protein domains
Figure S5 The Kaplan–Meier survival analysis for the five protein domains: Spectra, fn3, I-set, Ig_2, RhoGEF
Figure S6 Mutations in the ERBB2 signaling pathways.

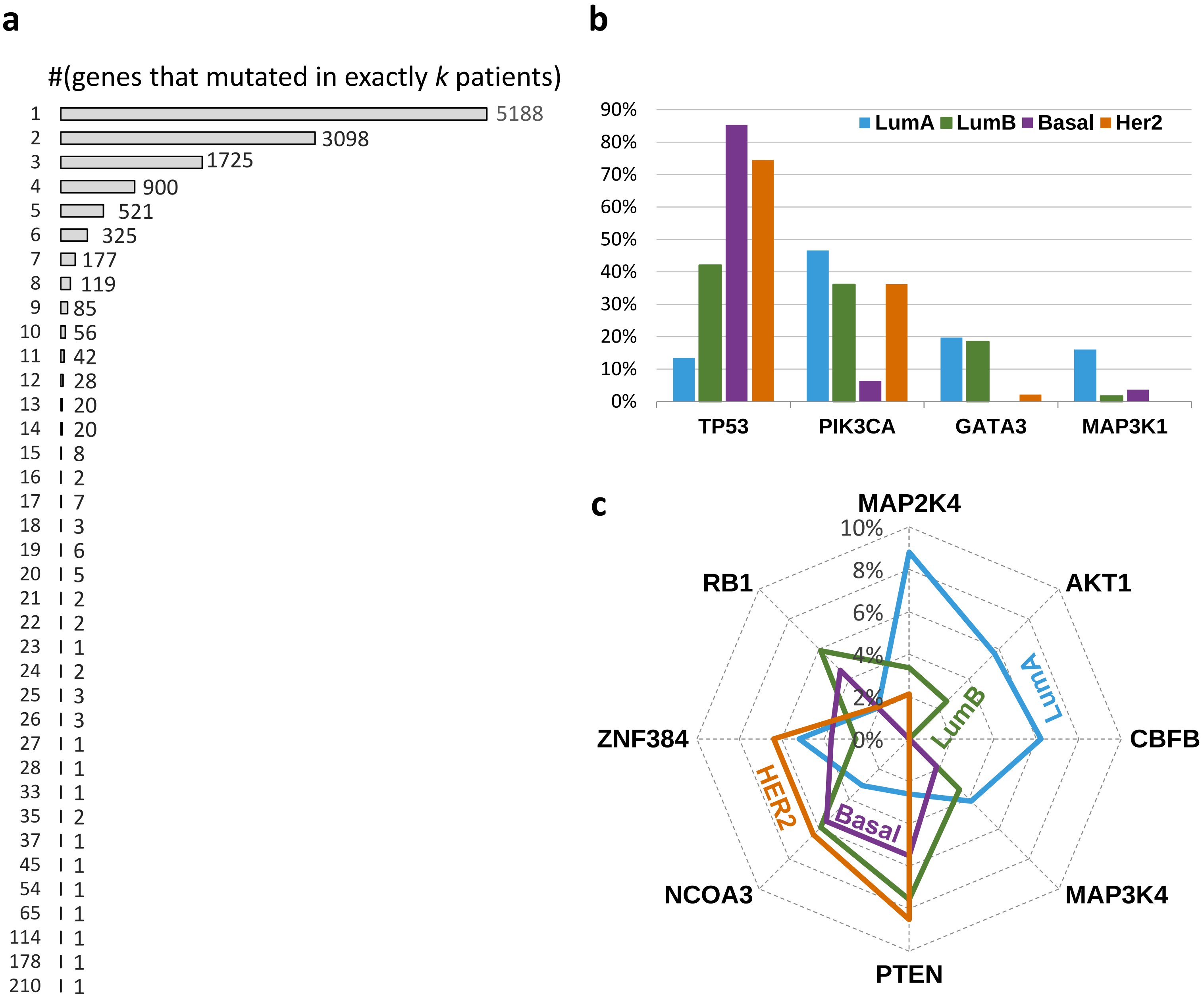

Figure S1 | (Related to Figure 1.) **The mutational heterogeneity of IDBC in terms of subtypes.**
(**a**) Bar chart for the number of mutated genes in a given number of patients.
(**b**) The mutation coverages (MCs) of the four significantly mutated genes with a mutation frequency > 9% in different subtypes.
(**c**) The MCs of the eight significantly mutated genes with a mutation frequency < 9% in different subtypes.

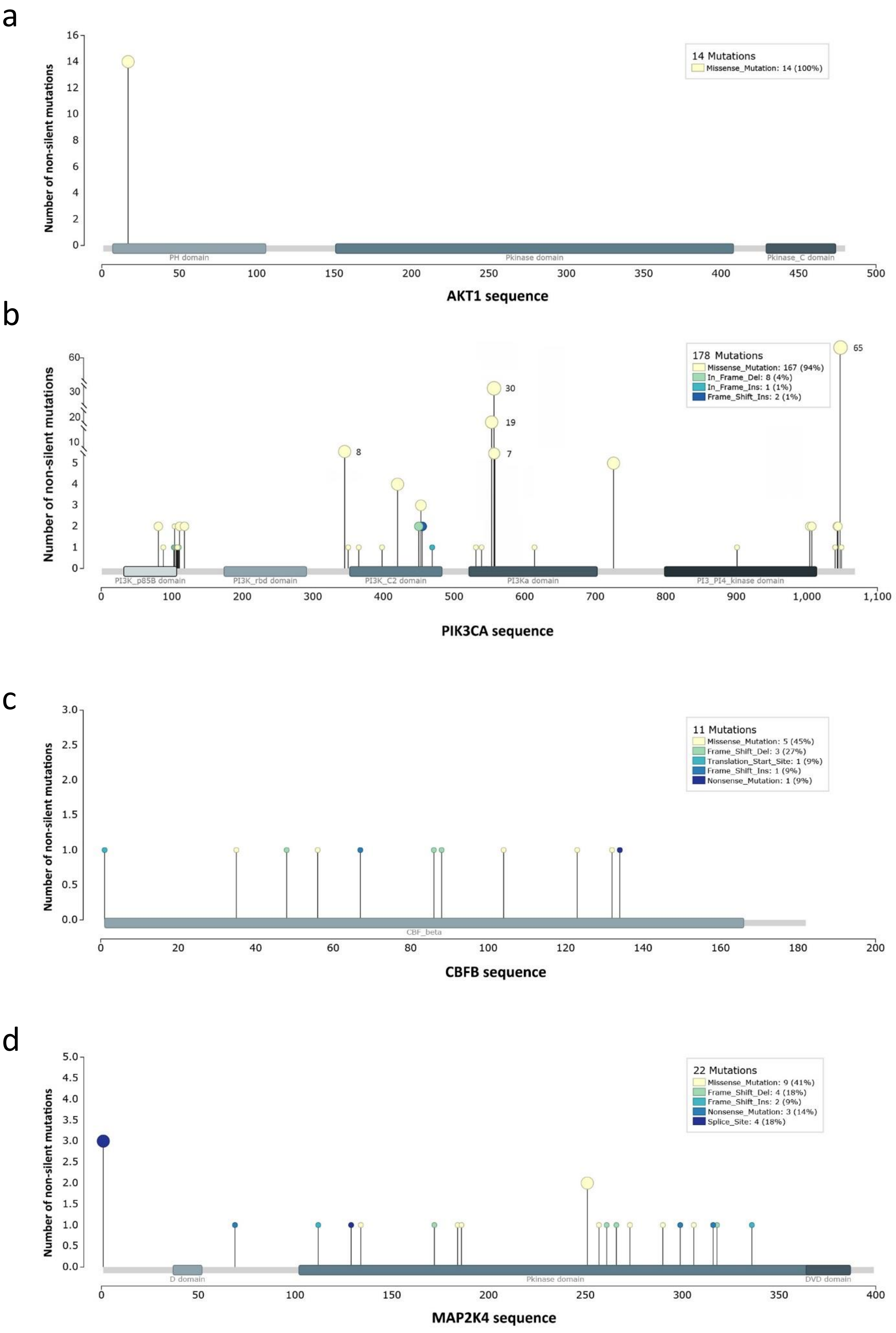

Figure S2 | (Related to Figure 1 and Table 1.) **Mutations in *AKT1*** (a), ***PIK3CA*** (b), ***CBFB*** (c) **and *MAP2K4*** (d).

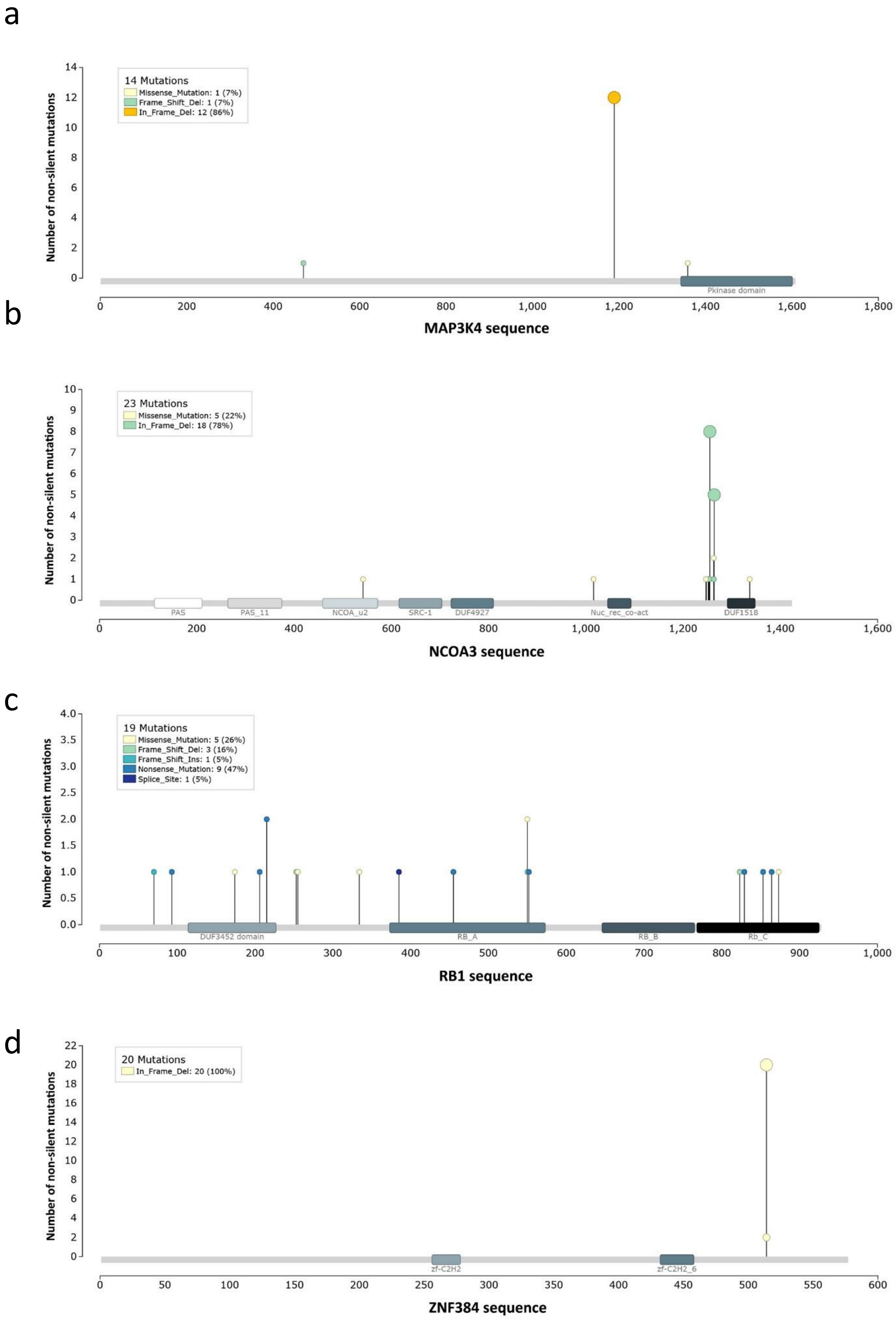

Figure S3 | (Related to Table 1.) **Mutations in *MAP3K4*** (a), ***NCOA3*** (b), ***RB1*** (c) **and *ZNF384*** (d).

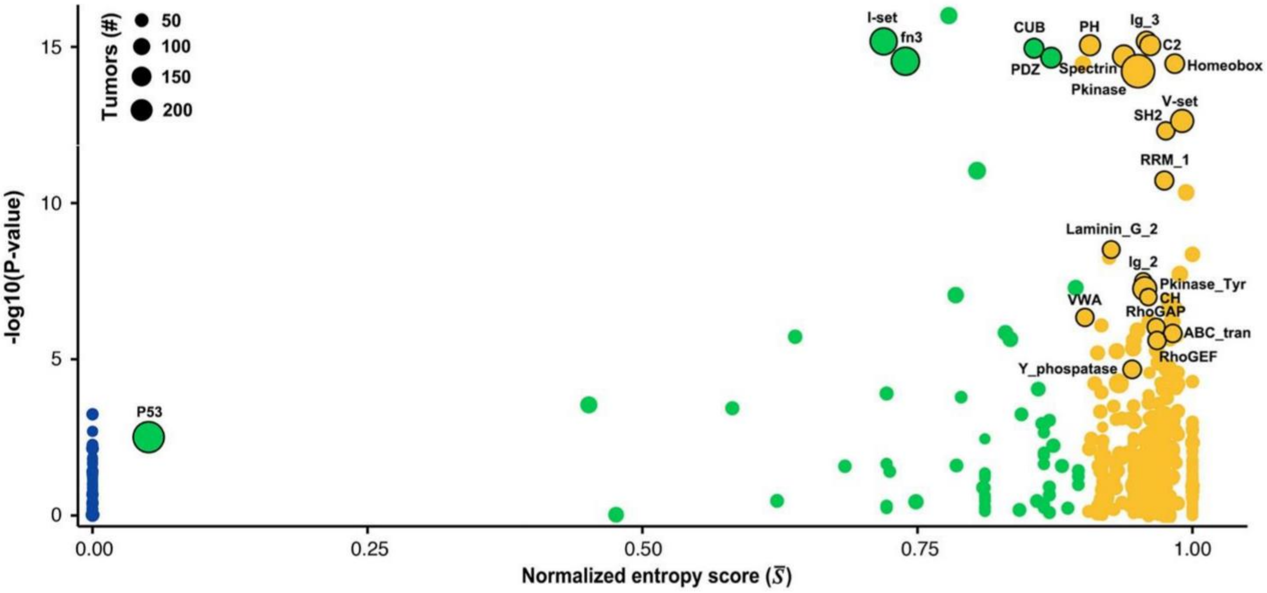

Figure S4 | (Related to Figure 2.) **-log(P-value) is plotted against the Shannon entropy ($\bar{S}$) for 1,217 protein domains.** The protein domains are colored blue, green and yellow for $\bar{S} = 0$, $0 < \bar{S} < 0.9$, and $\bar{S} \geq 0.9$, respectively. All labeled domains but P53 are those significantly mutated (P-value < 0.05, after Benjamini–Hochberg FDR adjustment) in at least 30 of the 468 patients.

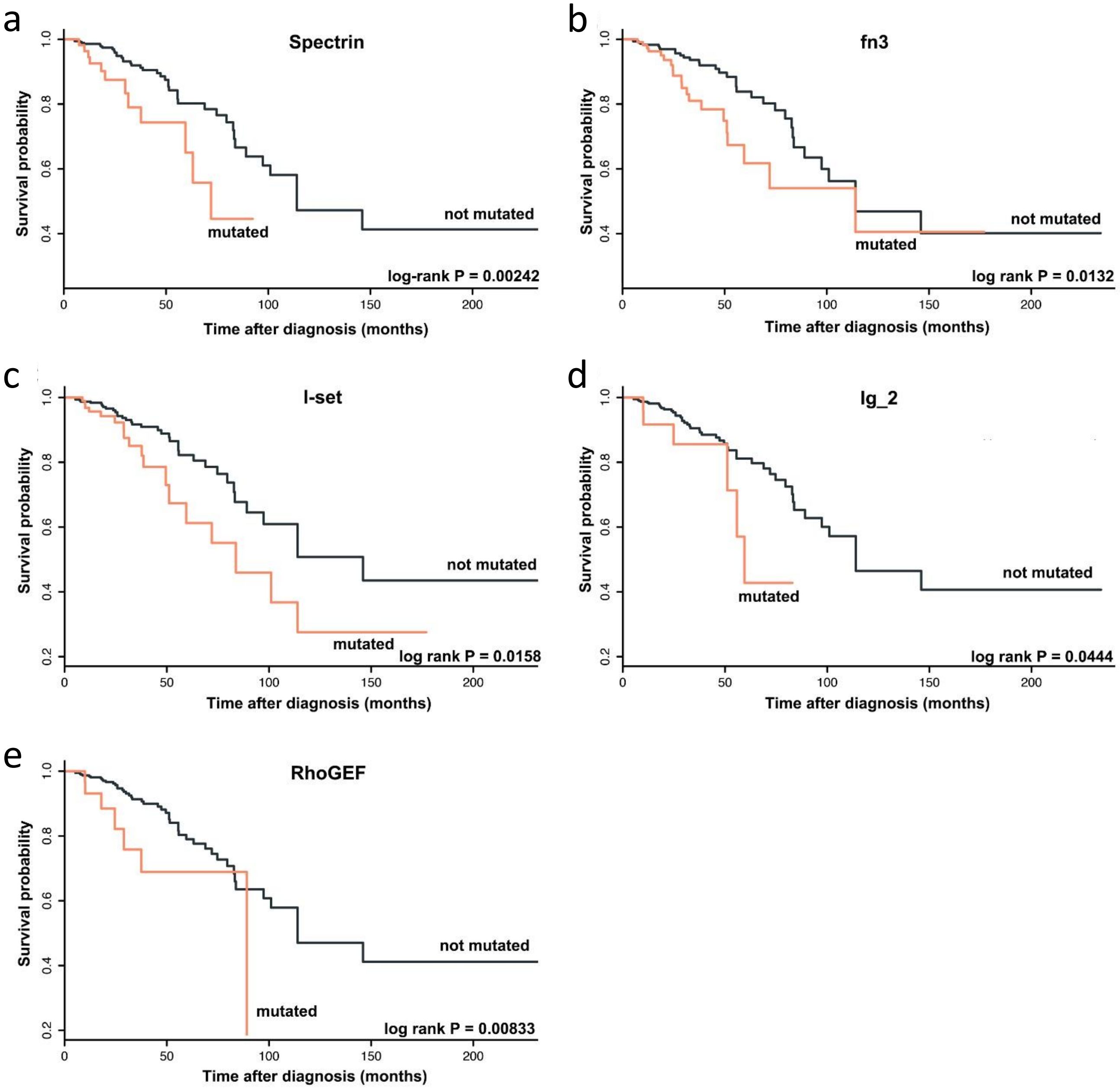

Figure S5 | (Related to Figures 2 and 3.) The Kaplan–Meier survival analysis for the five protein domains: Spectrin, fn3, I-set, Ig_2, RhoGEF.

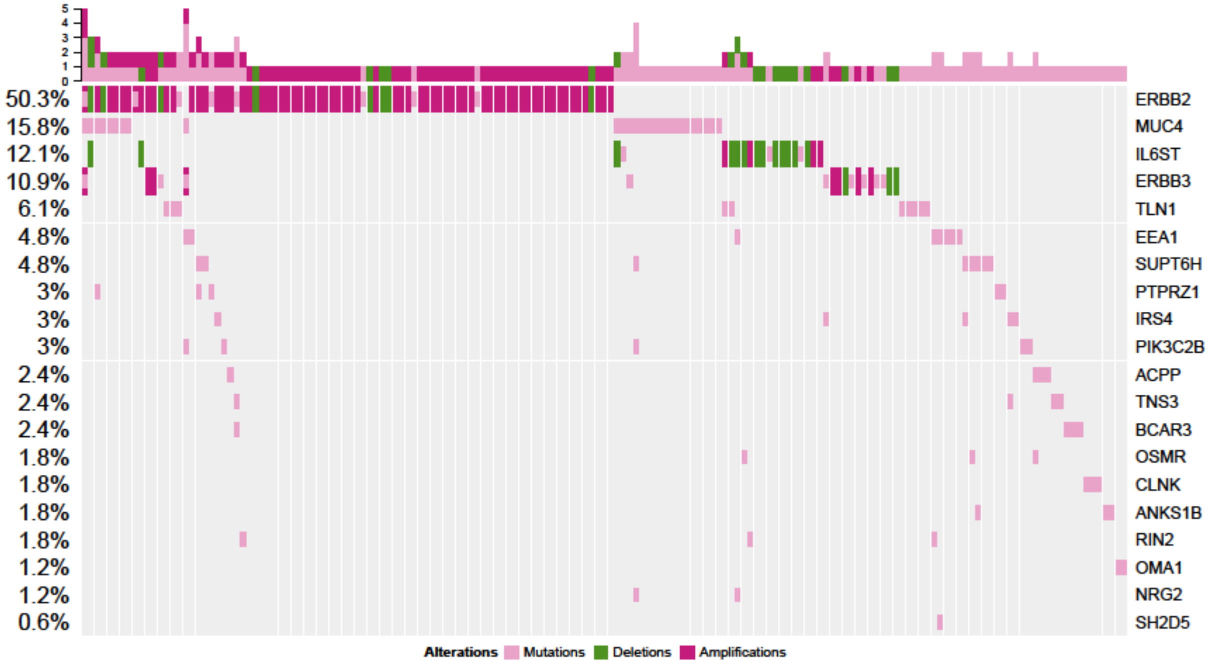

Figure S6 | Mutations in the ERBB2 signaling pathways.
